# X‑rays to Probe Compression across Scales in Rigid Polyurethane Foams: Molecular Simulations and Synchrotron Experiments

**DOI:** 10.1021/acsomega.6c01525

**Published:** 2026-04-27

**Authors:** Jacopo Lavazza, Jula C. Schroeder, Qicheng Zhang, Charles de Kergariou, Oier Bikondoa, Jemma L. Rowlandson, Tulio Hallak Panzera, Wuge H. Briscoe, Fabrizio Scarpa

**Affiliations:** † Bristol Composites Institute, 1980 University of Bristol, Bristol BS8 1TR, U.K.; ‡ School of Energy and Power Engineering, 121176 Beihang University, Beijing 100083, China; § XMaS, The UK CRG Beamline, European Synchrotron Radiation Facility (ESRF), Grenoble 38043, France; ∥ Department of Physics, 2707 University of Warwick, Coventry CV4 7AL, U.K.; ⊥ School of Electrical, Electronic, and Mechanical Engineering, University of Bristol, Bristol BS8 1TR, U.K.; # Centre for Innovation and Technology in Composite Materials, Department of Mechanical and Production Engineering, Federal University of São João del Rei, São João del Rei 36301-158, Minas Gerais, Brazil; ∇ School of Chemistry, University of Bristol, Bristol BS8 1TS, U.K.

## Abstract

Rigid polyurethane (PU) foams are widely used in engineering applications. This is underpinned by their multiscale and hierarchical structure, characterized by a nanometric segmented PU morphology and a closed-pore microstructure which determine the macroscopic material response. This work correlates the macroscopic behavior of rigid polyurethane foams derived from castor oil with their nanomorphological (in the crystalline domains) evolution upon compression. Molecular dynamics (MD) simulations were used to model the behavior of the PU chains in an ellipsoidal closed-pore structure, as well as the foam’s mechanical properties and X-ray diffractogram. Macro-scale experimental and MD simulation results were correlated using the Nagy model to account for strain rate effects. Synchrotron wide-angle X-ray scattering (WAXS) measurements were performed on the foams before and after compression, with the results compared with simulated diffractograms. A shift in the interplane spacing in the semicrystalline phase of the hard PU segments was observed upon compression, indicating a change in the nanostructure resulting from macroscopic mechanical loading.

## Graphical abstract

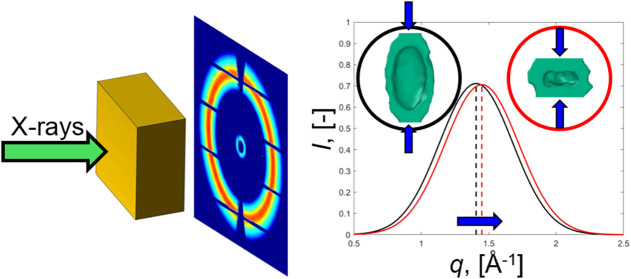

## 1 Introduction

Rigid polyurethane foams (RPUFs) are a class of porous polymer materials widely used in thermal insulation, packaging, construction, and the automotive industry.^1,2^ They are typically produced through the polyaddition reaction of polyols and isocyanates,^3^ allowing one to tailor their chemical structure. Moreover, the composition tunability allows a wide range of properties to be obtained, including low density,^4^ excellent compression strength,^5^ energy absorption,^6^ vibration damping^7^ and low thermal conductivity.^8^

Legislative requirements related to environmental protection and depletion of petroleum resources have led the industry toward new, sustainable solutions. Recent research has focused on developing biobased polyols from renewable sources, such as vegetable oils^9,10^ thanks to their abundance, low cost, biodegradability and ease of extraction.^11^ Castor oil is one of those renewable sources, and it is mainly composed by ricinoleic acid, which naturally presents a hydroxyl group in the C12 position.^12^ This enables the oil to be used directly as a polyol for synthesizing rigid polyurethane foams in combination with isocyanates—without requiring additional chemical functionalization, unlike other vegetable oils. As a result, it has gained widespread adoption as a sustainable, green material.^13^

The favorable properties of RPUFs result from their cellular structure, primarily consisting of closed pores containing trapped CO_2_ gas, a byproduct of the foaming reaction.^14^ Regarding the compressive behavior of foams, three typical response regimes are observed: linear elastic, plateau, and densification.^15^ The different regimes are associated with microstructural deformation mechanisms of the pores under compression, such as elastic bending,^16^ plastic buckling,^17,18^ and wall interaction.^19^ The control of the porosity, pore size and pore shape is fundamental to improving the mechanical performance of the foam.^20–22^

Although the macroscopic mechanical behavior of foams can be explained in terms of their microscopic characteristics, the macromolecular characteristics of polyurethane (PU) and their interactions at micro- and macro-length scales are less investigated. Generally, two distinct phases are observed in PU polymer: soft segments (SS)—constituted by polyols—and hard segments (HS)—isocyanates and chain extenders.^23^ The ratio between HS and SS typically controls the mechanical and physical properties of polyurethane, such as stiffness, thermal stability, and solvent interactions.^24–26^

Over the past few decades, small-/wide-angle X-ray scattering (SAXS/WAXS) has been extensively used to characterize the phase-separated nanostructure of PUs. Grünbauer and Folmer investigated RPUFs via SAXS and observed that the phase boundary and their self-similar nature were captured by the transition from monotonic to periodic intensity patterns.^27^ Creagh et al. described the semicrystalline nature of PU elastomers through in situ SAXS/WAXS measurements during tensile tests, noticing that crystallites were associated with the HS ordered structure, while amorphous regions and grain boundaries constituted SS.^28^ Similarly, Martin et al. developed a microdeformation stage to test PU foam struts during SAXS measurements. They observed anisotropic SAXS patterns associated with the preferential orientation of the HS crystallites and an increase of *d*-spacing during tensile tests.^29^ The results were confirmed by Laity et al., who observed the preferential alignment of the hard segments domains along the tensile loading direction via SAXS and proposed a globular model to describe the domain morphology.^30^ Furthermore, Kojio et al. performed SAXS/WAXS measurements during tensile testing of PU elastomers and observed additional diffraction peaks emerging with increasing strain, most likely related to strain-induced crystallization of the short segments.^31^

Previous findings indicate a close relationship between nanoscale morphology and macroscopic deformation in PUs, emphasizing the multiscale nature of RPUFs. However, previous in situ SAXS/WAXS studies have primarily focused on the tensile behavior of materials. In contrast, rigid polyurethane foams are typically employed as cores in sandwich panels and energy-absorbing structures, where their load-bearing capacity, crashworthiness, and damping properties are governed by their compressive response.^32,33^ This highlights the importance of this specific deformation mechanism for technological applications. Gebhart et al. showed the potential of a multiscale modeling in describing and predicting the behavior of expanded polypropylene foams by combining measurements of crystallinity, pore shape and size, and effective mechanical properties.^34^

Molecular dynamics (MD) simulations have proven to be an invaluable tool to model and understand the nanostructure of PU materials. Lempesis et al. developed in-depth atomistic simulations of the crystalline structure and mechanical response under tension of short segments,^35^ hard segments^36^ and of their combination in realistic PU copolymers.^37^ In addition, Ma et al.’s study on the fracture of PU materials through coarse-grained MD simulations revealed that fracture toughness increased with higher HS content.^38^ Wu et al. explored the use of MD simulations to represent the formation of a closed pore structure resembling those of RPUFs, and its behavior under compression.^39,40^

To the best of our knowledge, insights from SAXS/WAXS measurements and MD simulations have yet to be correlated in the open literature, highlighting a critical gap in understanding how deformation is a multiscale process in rigid polyurethane foams. This work aims to address this gap by establishing a correlation between macroscopic compression and nanostructural changes in rigid polyurethane foams using an innovative methodology.

Three classes of castor oil-based RPUFs have been previously subjected to quasi-static compression (QS) and drop weight-impact tests (low-velocity impacts, LVI).^41,42^ Synchrotron WAXS measurements were performed to observe the alterations of the nanoscale morphology upon compression. A full-atom, closed-pore molecular dynamics model was developed to capture the anisotropic mechanical response of the foams and simulate the corresponding diffractograms from this molecular configuration. In addition, the experimental and molecular dynamics mechanical responses were correlated through an empirical material model for porous materials, effectively bridging the strain-rate-dependent properties across length scales.

## 2 Methods

### 2.1 Materials and Specimen Preparation

Three castor oil (CO)-based RPUF formulations were evaluated in this work. Foam samples were obtained by mixing Mamonex RD70 A (RD A) isocyanate with CO additives, with two different polyester polyols derived from CO, namely Mamonex RD70 B (RD B) and Imperveg AGT 1315 B (AGT B). The mixes were carried out using the three different ratios listed in Table 1 and referred to as RF1, RF2, and RF3, respectively. All formulations were prepared by manually mixing polyols and isocyanate for about 1.5 min. The mixture was then poured into medium-density fibreboard molds (250 × 250 × 60 mm^3^) and cured for 14 days.^41,42^ All chemicals were supplied by Imperveg Polímeros Ind e Com (Brazil), and the foam samples were produced at the Federal University of São João del-Rei. No further details about additives used in the formulation were available from the supplier.

**Table 1:** Chemical Formulation of the Three Foams Characterized in the Work

foam	RD A, [wt %]	RD B, [wt %]	AGT B, [wt %]
RF1	60	40	0
RF2	50	20	30
RF3	50	40	10

Mechanical tests were performed on rectangular specimens measuring 30 × 30 × 15 mm^3^ in size obtained from hot-wire cutting. Five specimens were tested in each condition for statistical robustness. The mechanical properties of the foams were evaluated when loading the materials along the foam rising direction *d*_r_ and the transverse *d*_t_ direction, perpendicular to *d*_r_.

### 2.2 Mechanical Testing

#### 2.2.1 Quasi-Static Compression

The quasi-static compressive behavior of the foams was evaluated via monotonic loading, up to 80% nominal strain. Tests were performed using a Shimadzu AGS-X tabletop tester with a 1 kN SSM-DAM load cell at room temperature and with constant cross-head displacement of 2 mm min^–1^ (corresponding to a strain rate, ε̇, of 0.002 s^–1^). Further details can be found in ref 41.

#### 2.2.2 Low-Velocity Impact

The low-velocity impact (LVI) response of the foams was evaluated via drop impact tests performed using an Instron 9450 drop tower. The machine was equipped with a C-7529–302 strain gauge tup (or impactor) with a capacity of 45 kN and a C-7529–350 flat-faced circular insert (diameter of 50 mm). The impact force signal was obtained from the strain gauge tup, whereas the displacement signal was recorded via a photocell. The total drop mass was 5.6 kg. Four different impact energies (*J*_i_) corresponding to 5, 10, 20, and 30 J were applied by varying the drop height of the weight. The drop weight velocity before impact (*v*) was measured by the photocell. The velocity and strain rate corresponding to each *J*_i_ are reported in Table 2. Further details can be found in ref 42.

**Table 2:** Impact Velocity (*v*) and Strain Rate (ε̇) for the Different Impact Energies (*J*_i_)

*J*_i_, [J]	*v*, [m s^–1^]	ε̇, [s^–1^]
5	1.33	87 ± 2.91
10	1.88	123 ± 1.83
20	2.66	174 ± 5.01
30	3.26	214 ± 6.88

#### 2.2.3 Foam Mechanics Parameters

The mechanical behavior of the foams was described in terms of nominal stress (σ) and nominal strain (ε). In this work, σ and ε are considered positive in compression.

The apparent density (ρ) was calculated from the mass (*m*_s_) and volume (*V*_s_) of foam specimens according to eq 1

1 $$\rho =\frac{m_{s}}{V_{s}}$$

The specimens’ dimensions were measured using a Siegen digital electronic vernier calliper with an accuracy of ±0.01 mm and masses were measured with a Mettler Toledo XS203S XS balance with an accuracy of ±0.001 g.

The material response in the linear elastic region was quantified by the compressive modulus (*E*), calculated as the tangent modulus at ε → 0% (from the linear fitting of the region 0% ≤ ε ≤ 5%) from eq 2

2 $$E={\frac{d\sigma \left(\varepsilon \right)}{d\varepsilon }|}_{\varepsilon \to 0\%}$$

The energy absorption efficiency (efficiency, *U*) has been used to evaluate the energy absorption characteristics of porous materials,^43,44^ and is defined in eq 3

3 $$U=\frac{1}{\sigma \left(\varepsilon \right)}\int_{0}^{\varepsilon }\sigma \left(\varepsilon \right)d\varepsilon$$

The strain at densification (*ε*_d_) represents the upper limit of the plateau region. It is defined as the strain at which the energy absorption efficiency reaches a maximum,^45^ satisfying eq 4

4 $${\frac{dU}{d\varepsilon }|}_{\varepsilon =\varepsilon_{d}}=0$$

The plateau stress (σ_pl_) characterizes the plateau region and is defined from eq 5 ^46^

5 $$\sigma_{p}=\frac{1}{\varepsilon_{d}-\varepsilon_{y}}\int_{\varepsilon_{y}}^{\varepsilon_{d}}\sigma \left(\varepsilon \right)d\varepsilon$$

In ([Disp-formula eq5]), *ε*_y_ is the strain corresponding to the yield stress (σ_y_), which identifies the upper limit of the linear elastic region. The yield point was identified using the tangent method reported by Viot previously,^47^ with manual checks confirming consistent results. The peak stress (σ_max_) is defined as the maximum stress reached during loading.

### 2.3 Wide-Angle X-ray Scattering

#### 2.3.1 Experimental Setup

Wide-angle X-ray scattering (WAXS) measurements were carried out on the BM28 beamline at the European Synchrotron Radiation Facility (ESRF) in Grenoble, France.^48^

The experimental setup is shown in Figure 1. The experiments were conducted in a transmission geometry using a photon energy of 15 keV, corresponding to a wavelength λ = 0.827 Å, with a beam size of 80 μm × 90 μm (width × height). Measurements were performed with a pixel array detector, Pilatus 1 M (Dectris Ltd.), with a sample-to-detector distance of 0.31 m, covering a momentum transfer (*q*) range of 0.12–2.85 Å^–1^. Measurements were performed on foam specimens subjected to LVI at different impact energies (3 days after impact testing) and on virgin specimens along the rise and transverse direction. Three separate samples were used per experimental condition, and five measurements were performed for each sample at five different spots, 1 mm apart along the horizontal direction, with an integration time of 10 s for each measurement.

**Figure 1:**
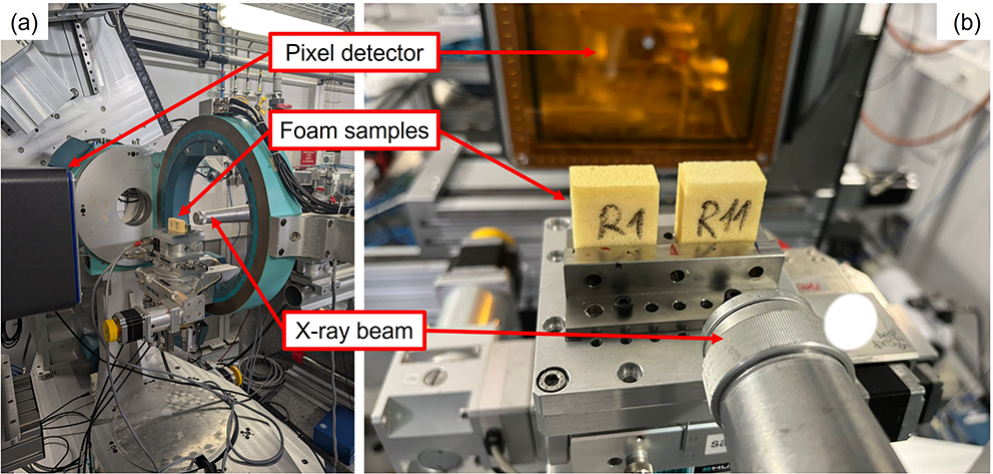
Wide-angle X-ray scattering (WAXS) experimental setup: (a) side view and (b) front view with a focus on the samples.

#### 2.3.2 Analysis and Statistical Treatment of the Data

The recorded WAXS 2D data were normalized by transmission, with background subtracted, and azimuthal regrouping (azimuthal range 0° ≤ χ ≤ 90°) was performed to obtain 1D WAXS profiles using the PyFAI software.^49,50^ The raw data sets are available at the ESRF repository.^51,52^ The MATLAB function anova2 was used to perform two-way analysis of variance (ANOVA), and therefore to compare the statistical performance of measured data with respect to foam directions (*d*_r_ and *d*_t_) and impact energies (*J*_i_ = 0, 5, 10, 20, 30 J).

### 2.4 Molecular Dynamics (MD) Simulations

#### 2.4.1 Model Setup and Pore Formation

The PU macromolecular structure investigated in this work is shown in Figure 2(a). The soft segment was a poly-(tetramethylene oxide) (PTMO), while the hard segment was a 4,4′-diphenylmethane diisocyanate (MDI) with *n*-butanediol (BDO) as the chain extender. The structure has already been extensively investigated in PU molecular simulations in literature.^35–38,53^

**Figure 2:**
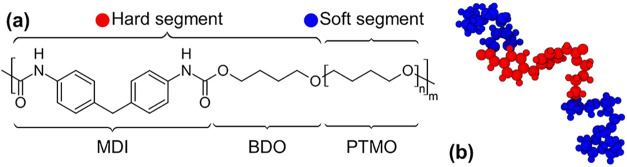
(a) Chemical formula of the polyurethane repeat unit considered in this work (*n* = 8 and *m* = 1); (b) 3D visualization of a polyurethane molecule, highlighting the hard (red) and soft (blue) segments.

MD simulations were performed in the Large-scale Atomic/Molecular Massively Parallel Simulator (LAMMPS),^54^ with the polymer-consistent force field (PCFF)^55^ adopted to describe the PU chains, which has been employed for various polymeric systems including PU.^38,56–58^ The initial topology of the system and the default PCFF parameters were generated using the Enhanced Monte Carlo (EMC)^59^ package. Three-dimensional visualization was rendered using the Open Visualization Tool OVITO.^60^ A total of 100 PU chains (shown in Figure 2(b)) were generated per simulation, resulting in a system of 15700 atoms. Ten independent initial configurations were generated to achieve statistical significance. During simulations, a time step of 1 fs was used within the velocity Verlet algorithm to perform time integration and the long-range cutoff distance was set to 10 Å using the Particle–Particle Particle-Mesh (PPPM) method.

The system underwent a multistep equilibrium process. First, it was equilibrated at a high temperature (600 K) in a canonical (NVT) ensemble, employing the Nose–Hoover thermostat for 15 ps (see Figure 3(a)). Subsequently, a closed pore was formed based on the methodology proposed by Wu et al.^39,40^ Two spherical surfaces were created with initial a radius of *R*_i_ = 0.1 nm (the inner pore surface) and *R*_o_ = 15 nm (the outer pore surface), respectively, while the system was still under NVT and at 600 K. The outer radius was reduced from 15 to 4 nm while the inner radius was enlarged from 0.1 to 2.5 nm over 10 ps (shown in Figure 3(b)). To reproduce the anisotropic nature of the RPUFs, the pore was elongated into an ellipsoidal shape, with the rise direction (*d*_r_) matching the *Z* axis of the simulation (see Figure 3(c)). Two pore models were constructed with the anisotropy ratio (*c*/*a*, the ratio between major and minor axes) of 1.5 and 2, which are the bounding values of the experimental *c*/*a* observed (see Supporting Information). The pore dimensions for the two systems are reported in Table 3.

**Figure 3:**
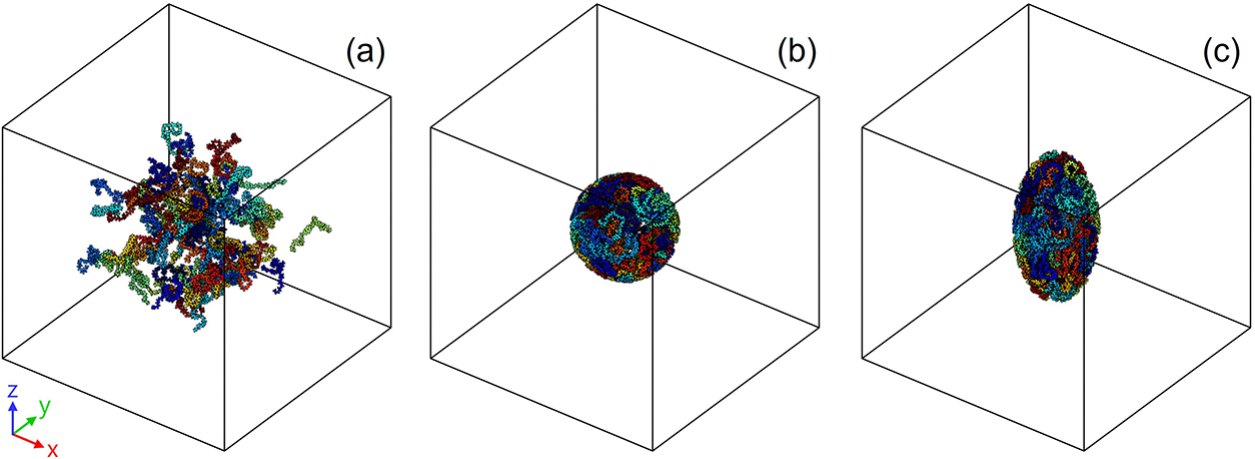
3D visualization of the molecular system: (a) equilibrated system; (b) spherical pore; and (c) ellipsoidal pore (*c*/*a* = 2).

**Table 3:** Anisotropy Ratio (*c*/*a*) of the Two Pore Models Constructed and Values of Major Outer Shell Axis (*c*_o_), Minor Outer Shell Axis (*a*_o_), Major Inner Shell Axis (*c*_i_), Minor Inner Shell Axis (*a*_i_)

*c*/*a*, [-]	*c*_o_, [Å]	*a*_o_, [Å]	*c*_i_, [Å]	*a*_i_, [Å]
1.5	52.5	35	34.5	23
2	64	32	40	20

The pore shape transitioned from a hollow sphere to a hollow ellipsoid over 10 ps under NVT at 600 K. The system was finally cooled from 600 to 300 K to release internal stresses under an isobaric ensemble (NPT) using the Nose–Hoover barostat. The pressure was then lowered from 100 to 1 atm to reach ambient conditions.

#### 2.4.2 Compression Simulations

The PU ellipsoidal pores were deformed under uniaxial compression at a constant strain rate of 10^10^ s^–1^ (comparable with typical strain rates used in relevant literature^61,62^ ) under NPT ensemble at 300 K, ensuring a zero-pressure condition for the two lateral simulation cell faces. Compression was simulated until a prescribed nominal strain of 70%, along the *d*_r_ and *d*_t_ directions. The rise direction is the *Z* axis in Figure 3, while the transverse one is represented by the *X* axis in Figure 3. The two directions were considered to investigate the physical and mechanical effects of the pore anisotropic shape.

While the strain rate in MD simulations (10^10^ s^–1^) is necessarily much higher than experimental rates due to computational constraints, the Nagy model was used to bridge this gap (see Supporting Information).

#### 2.4.3 Diffraction Simulations and Analysis

Diffraction simulations were performed using the method developed by Coleman et al.^63^ 1D X-ray diffraction (XRD) were calculated before and after the compression simulation of the system.

The mesh spacing (in the reciprocal space) was set to 0.009 Å^–1^ and the wavelength (λ) was set to 1.54 Å (simulating a Cu Kα source). The XRD was computed over the 5° ≤ 2θ ≤ 100° interval. For comparison with experimental WAXS data, the 2θ angle was converted to momentum transfer (*q*) using eq 6

6 $$q=\frac{4\pi }{\lambda }\sin\left(\frac{2\theta }{2}\right)$$

The diffraction intensity (*I*) at each reciprocal lattice point was computed using kinematic diffraction theory, as in eq 7

7 $$I\left(q\right)=\frac{F\left(q\right)F^{*}\left(q\right)}{N}$$

where *N* is the number of atoms in the system, and *F*(**q**) and F*­(**q**) are the structure factor and its complex conjugate. The structure factor is defined in eq 8, where the atomic scattering factors (*f*_*j*_(**q**)) are defined from analytical approximations parametrized for most elements. Further details can be found in the original work.^63^

8 $$F\left(q\right)=\sum_{j=1}^{N}f_{j}\left(q\right)\exp\left(iq\cdot r_{j}\right)$$

## 3 Results and Discussion

### 3.1 Compressive Behavior across Scales

The RPUF’s porous structure and compressive behavior were simulated through molecular dynamics (MD) as described above. Ten independent initial configurations were tested for statistical significance and to reduce noise in the results. The mean response was calculated by averaging the raw data from individual simulations and is discussed in the following section.

The nominal stress–strain (σ–ε) curves obtained from the pore compression simulations are reported in Figure 4 for the rise (*d*_r_) and transverse (*d*_t_) direction, alongside the visualization of the pore structure evolution during the process. The curves follow the experimental compressive behavior of cellular plastics, presenting a linear elastic, plateau and densification region.^15^ While the curve along *d*_r_ strongly resembles the experimental stress–strain response (see Supporting Information), the simulated response along *d*_t_ exhibits more limited plateau regions, with densification occurring at ε = 30.1% (*c*/*a* = 1.5) and ε = 34.3% (*c*/*a* = 2). This phenomenon is related to the loss of the original porous structure in the system, as highlighted in 3D visualizations in Figure 4. In contrast, the system still presents an empty pore at large strains when compressed along the rise direction. Therefore, when compressed along *d*_t_, the pores collapse early, and the material enters a densified regime, leading to a rapid increase in stress at lower strains compared to *d*_r_.

**Figure 4:**
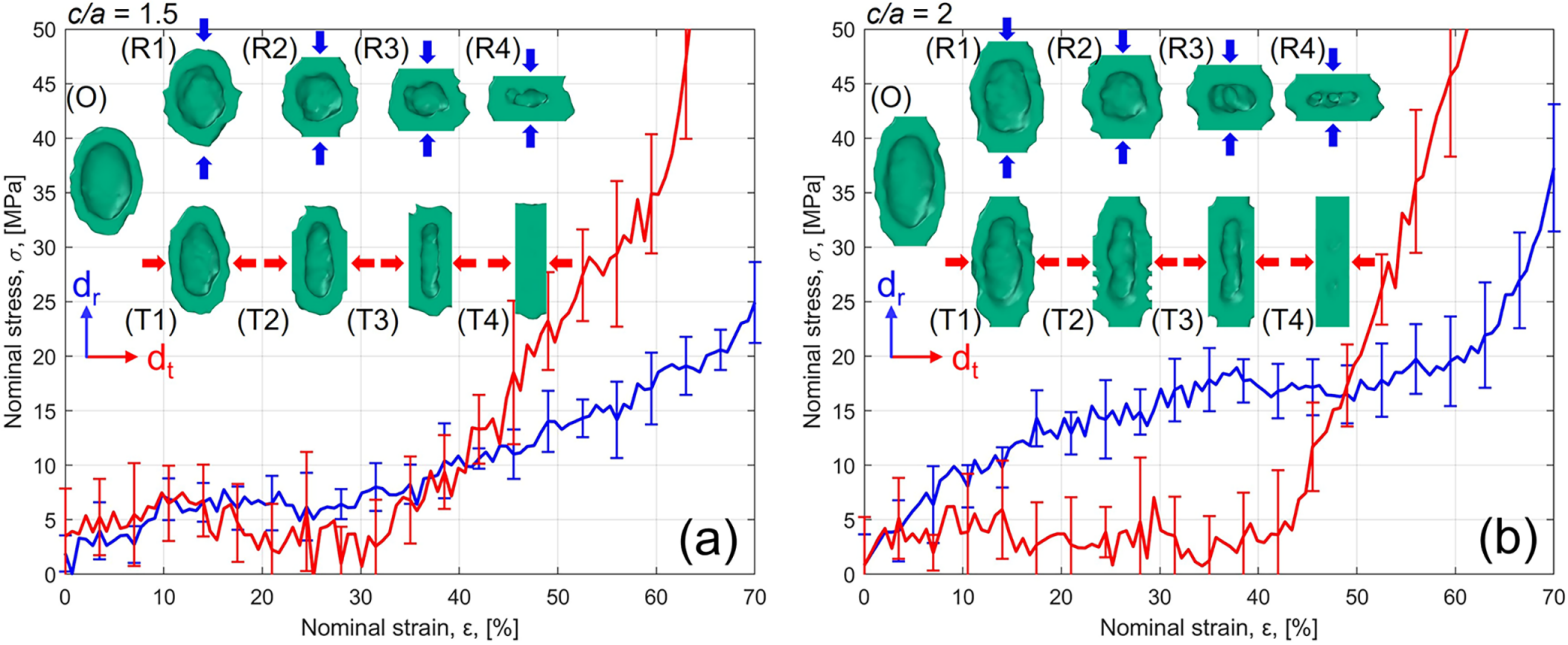
Nominal stress–strain (σ–ε) curves for simulated pore compression along the rise (*d*_r_, blue) and transverse (*d*_t_, red) direction with (a) *c*/*a* = 1.5 and (b) *c*/*a* = 2. 3D visualizations (green surfaces, obtained in OVITO) of the pore cross-section and deformation during compression: (O) ε = 0%, (R1–T1) ε ≈ 15%, (R2–T2) ε ≈ 35%, (R3–T3) ε ≈ 50%, (R4–T4) *ε* ≈ 60%.

The characteristic compressive mechanical properties were calculated as described above and are summarized in Table 4.

**Table 4:** Average Mechanical Properties Obtained from the Molecular Dynamics Compression Simulation in the Rise (*d*_r_) and Transverse (*d*_t_) Direction, for Different Anisotropy Ratios (*c*/*a*)­^a^

*c*/*a*, [-]	direction	*E*, [MPa]	σ_y_, [MPa]	σ_p_, [MPa]	σ_max_, [MPa]	ε_y_, [%]	ε_d_, [%]
1.5	*d*_r_	32.1	5.8	10.6	24.9	13.3	64.4
	*d*_t_	21.1	7.2	4	131.1	11.5	30.1
2	*d*_r_	90.4	9.6	15.6	37.3	9.4	60.9
	*d*_t_	28.8	2.9	3.5	146.6	6.7	34.3

a Compressive modulus (*E*), yield stress (σ_y_), plateau stress (σ_p_), peak stress (σ_max_), yield strain (ε_y_) and densification strain (ε_d_).

The pores exhibit a highly anisotropic mechanical response, with higher performance along the rise direction and for *c*/*a* = 2. This result provides a significant insight into the relation between pore morphology and compressive behavior, as the observed anisotropy solely originates from the ellipsoidal shape of the molecular system itself. This phenomenon is in agreement with experimental results^41,42^ and can be attributed to the buckling resistance of the pore cross-section. With a mundane analogy to eggs,^64^ when the pore is loaded along the rise direction, the cross-section is circular. It provides higher stiffness and stability to the structure compared to the elliptical cross-section when compression occurs along the transverse direction.

To further confirm the role of the pore architecture on the mechanical response, a direct comparison with experimental results is required. On the other hand, the strain rate (ε̇) employed during MD compression simulations was set to 10^10^ s^–1^ because of the reduced characteristic times used in MD simulations, which are in the order of fs. Currently, such strain rates cannot be achieved experimentally even with advanced systems like Split–Hopkinson pressure bars, which are still limited to around 10^4^ s^–1^.^65,66^ Extrapolation methods based on the time–temperature equivalence and shifting have been proposed to predict the accuracy of the results at MD strain rates.^67^ However, they would require extensive simulations at different temperatures to obtain the material master curve.

Nevertheless, the strain rate effect in polymeric cellular materials has been extensively investigated and modeled during the past decades. Nagy et al. proposed a phenomenological constitutive model to account for strain rate effects,^68^ following eq 9

9 $$\sigma \left(\varepsilon \right)=\sigma_{0}\left(\varepsilon \right){\left(\frac{\mathrm{\varepsilon ̇}}{{\mathrm{\varepsilon ̇}}_{0}}\right)}^{a+b\varepsilon }$$

In eq 9, σ_0_(ε) is the reference stress–strain curve, ε̇ is the strain rate, ε̇_0_ is the reference strain rate and *a* and *b* are coefficients identified by inverse fitting. In the original work, Nagy et al. limited the experimental strain rate range to 10^–3^ ≤ ε̇ ≤ 10^2^ s^–1^, but it has since been shown that eq 9 can be used to predict strain rate effects up to 5 × 10^3^ s^–1^.^69^

Therefore, quasi-static (QS, ε̇ = 0.002 s^–1^)^41^ and low-velocity impact (LVI, ε̇ = 215 s^–1^)^42^ experimental average curves were used to calibrate the fitting parameters in eq 9 for the three RPUFs previously investigated (see Table 1) along the rise and transverse directions. Note that in LVI experiments, the strain rate can be assumed constant only until densification (ε ≤ 60%), as the impact velocity quickly drops at higher strains.^70^ The Nagy model was then used to predict the material response at ε̇ = 10^10^ s^–1^, allowing for comparison against the MD simulations. Relevant mechanical properties are reported in Table 5, and further details are provided in the Supporting Information.

**Table 5:** Mean Mechanical Properties Obtained from the Nagy Model (Equation 9) at Strain rate ε̇ = 10^10^ s^–1^ for the Three Foams (RF1, RF2, RF3, see Table 1) in the Rise (*d*_r_) and Transverse (*d*_t_) Direction^a^

foam	direction	*E*, [MPa]	σ_y_, [MPa]	σ_p_, [MPa]	ε_y_, [%]	ε_d_, [%]
RF1	*d*_r_	23.4	0.97	0.88	4.4	56.4
	*d*_t_	17.9	0.73	0.94	4.5	54.1
RF2	*d*_r_	89	4.1	5	4.3	54.8
	*d*_t_	70	3	5.2	4.8	47.3
RF3	*d*_r_	19.8	0.93	0.89	5.2	56.3
	*d*_t_	9.63	0.45	0.8	5.4	51.9

a Compressive modulus (*E*), yield stress (σ_y_), plateau stress (σ_p_), yield strain (ε_y_) and densification strain (ε_d_).

By comparing Tables 4 and 5, it is noticeable how the properties predicted from the Nagy model for the RF2 foam are on the same order of magnitude of those obtained from the MD simulation, with experimental values in between the numerical ones obtained for *c*/*a* = 1.5 and *c*/*a* = 2. The differences can be attributed to the bulk effects that emerge during the experimental compression of foam samples. Real RPUFs are constituted of multiple closed pores, which interact with each other during compression and limit the effective deformation of individual pores, transferring stress across the entire foam sample.^71^ On the other hand, the MD model presented in this work entails a single, isolated pore and, therefore, cannot capture this behavior.

It has been shown how the sphericity (i.e., how spherical a pore is) is a fundamental parameter in determining the mechanical properties of foamed materials.^72,73^ The measured *c*/*a* ratios for three foams (see Supporting Information) range from 1.5 to 1.9, in between those used in MD simulations of 1.5 and 2. A parametric study of the *c*/*a* ratio, could help reduce the gap between experimental and molecular dynamics (MD) results. In addition, the PU structure was fixed during the study because the LAMMPS integration of ellipsoidal regions is likely to introduce singularities that cause the loss of atoms during simulations.^74^ Simulating an ellipsoidal pore for long and complex macromolecules is challenging due to their reduced rotational freedom, which can cause the simulation to fail. A possible alternative would be represented by the use of coarse-grained models, which allow for the reduction of the number of particles in the system through the calculation of effective properties and have already been employed to simulate spherical pores.^75^ On the other hand, these models prevent the simulation of X-ray diffractions, in its current LAMMPS implementation. Those simulations require full-atom models to compute the scattering factors.

Nonetheless, the results presented in this work clearly indicate the value of correlating the compressive response of RPUFs across length scales based on simple implementations of phenomenological models, such as the Nagy model discussed above and MD simulations.

### 3.2 Correlating Diffraction Patterns to Deformation

Wide-angle X-ray scattering (WAXS) was employed to experimentally probe the nanostructure of three castor oil-based RPUFs (see Table 1) previously investigated by some of the Authors here.^41,42^ Specimens subjected to low-velocity impact at different energies (*J*_i_, see Table 2) and pristine specimens were investigated via WAXS. Average 2D maps and 1D signals obtained via azimuthal regrouping for the three RPUFs in the rise *d*_r_ direction are reported in Figure 5, for *J*_i_ = 0 J (pristine specimens) and *J*_i_ = 30 J (the highest impact energy value).

**Figure 5:**
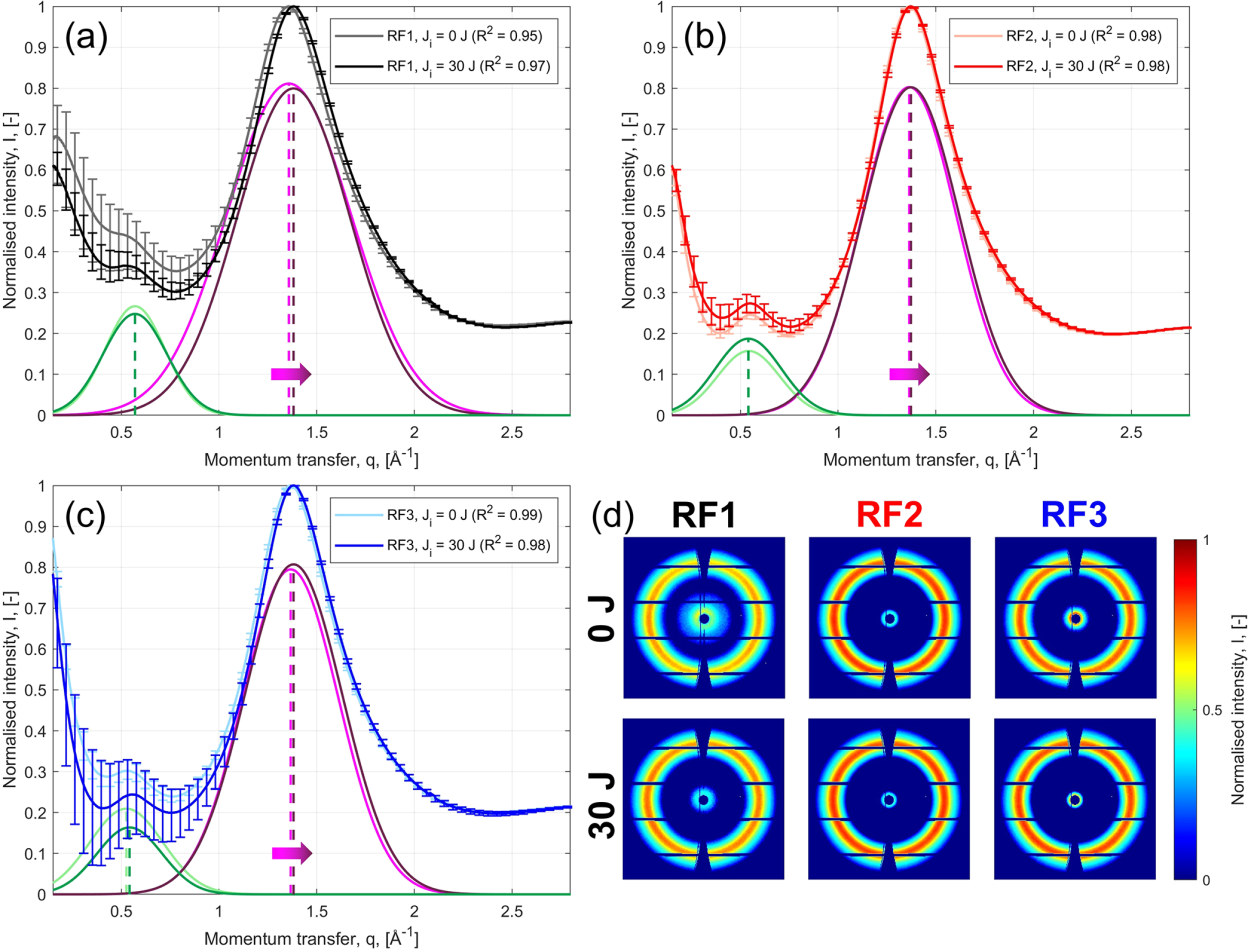
Experimental WAXS 1D signals for the three foams (see Table 1) in the rise (*d*_r_) direction before (*J*_i_ = 0 J) and after (*J*_i_ = 30 J) impact: (a) RF1, (b) RF2, (c) RF3. The primary (magenta) and secondary (green) peaks were obtained via fitting of Gaussian functions to the 1D data. (d) Experimental 2D WAXS maps. The color bar refers to the normalized intensity magnitude.

Isotropic patterns are observed in the 2D intensity maps, and diffraction rings appear uniform along the azimuthal angle χ. The diffraction peaks appear broad and diffused, indicating the PU material to be predominantly amorphous (given by the soft segments, SS) with limited crystalline domains in the hard segments (HS).^76^ The curves present two peaks at *q*_1_ ≈ 1.3 Å^–1^ (the primary peak) and *q*_2_ ≈ 0.5 Å^–1^ (the secondary peak), which were identified through Gaussian fitting.

However, diffraction patterns from MD simulations did not display the secondary peak, corresponding to supramolecular ordering of hard segment aggregates.^77–79^ This discrepancy likely arises not only from the relatively short PU chains simulated, which limit the development of long-range hard segment correlations, but also from the inability of the simulations to fully capture chain branching, phase segregation, and the distinct intradomain organization present in real foams. Therefore, the results and discussion will focus on the primary peak, in particular its intensity (*I*_1_) and position (*q*_1_). The values of *I*_1_ do not present a monotonic trend with increasing impact energy (*J*_i_), although in most cases the minimum intensity is observed at *J*_i_ = 20 J, as shown in Figure 6. The obtained ANOVA tables are reported in the Supporting Information. The parameters indicate that *I*_1_ values at different *J*_i_ are statistically different, confirming a nonmonotonic trend.

**Figure 6:**
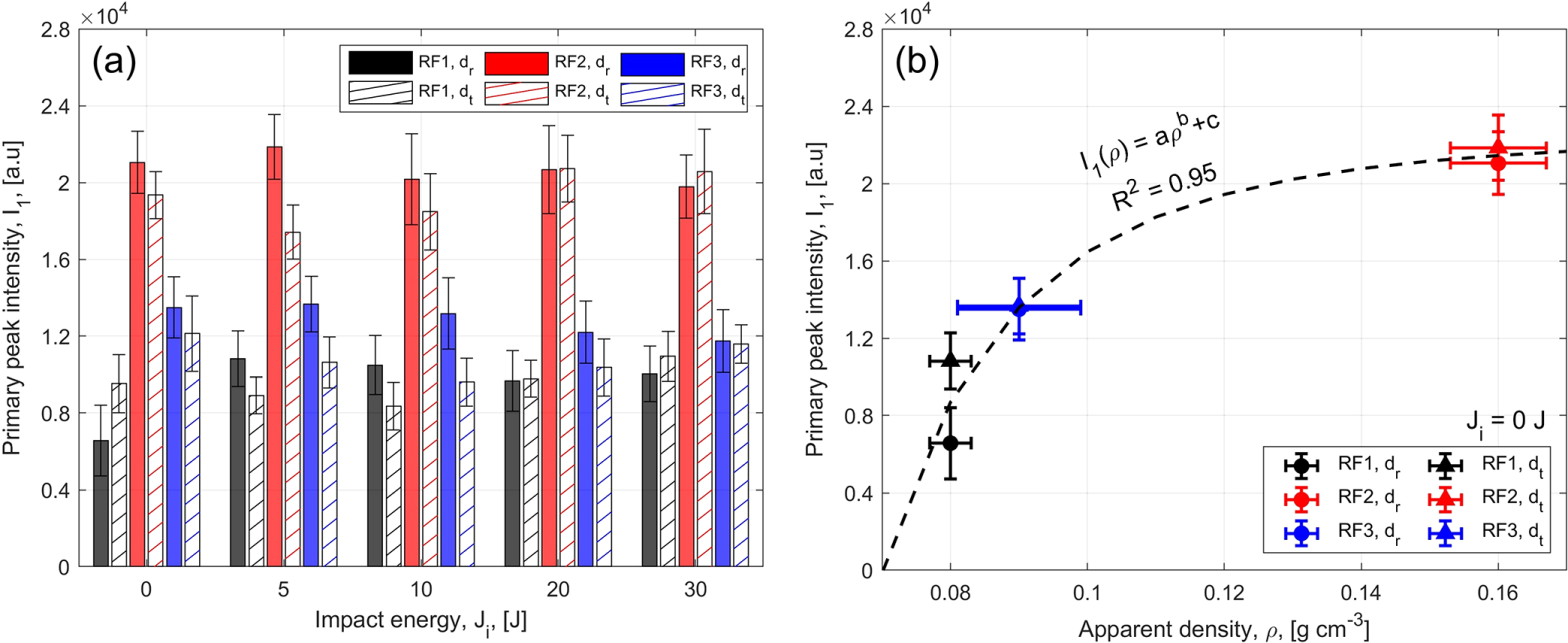
Variation of primary peak intensity (*I*_1_) with (a) impact energy (*J*_i_) and (b) foam apparent density (ρ, for *J*_i_ = 0 J). Values obtained via fitting of Gaussian functions to the 1D data of the three foams (RF1, RF2, RF3, see Table 1) in the rise (*d*_r_) and transverse (*d*_t_) direction.

On the other hand, the *I*_1_ value of the pristine sample has a power law dependency versus the apparent density of the foam, as shown in Figure 6(b). The power law has *a*, *b*, and *c* as fitting parameters. This trend was previously observed through lab-based powder X-ray diffraction in a study by some of the Authors here.^41^ In general, the intensity of the diffraction peak is related to the relative amount of phases in the system.^80^ As there is only one solid phase in the RPUFs (i.e., the PU polymer), the *I*_1_ value refers to the amount of material probed and, in turn, to the foam’s apparent density.

In addition, a distinct shift of the peak position *q*_1_ to higher values is observed for increasing *J*_i_, as shown in Figure 7. The tables with the ANOVA results are reported in the Supporting Information. For the three types of foams, low *p*-values (lower than 0.05) and high *F*-values indicate that *q*_1_ is strongly affected by the impact energy and the loading direction, as further emphasized by the presence of non-negligible interaction terms. To the best of the Authors’ knowledge, such nanostructure variations have not been documented in amorphous polymeric foams. The available literature has focused on the effect of tensile loading on the PU nanoscale morphology.^27–31^ Conversely, *q* shifts during loading have been described both experimentally and computationally for a variety of amorphous and crystalline inorganic materials.^81–83^

**Figure 7:**
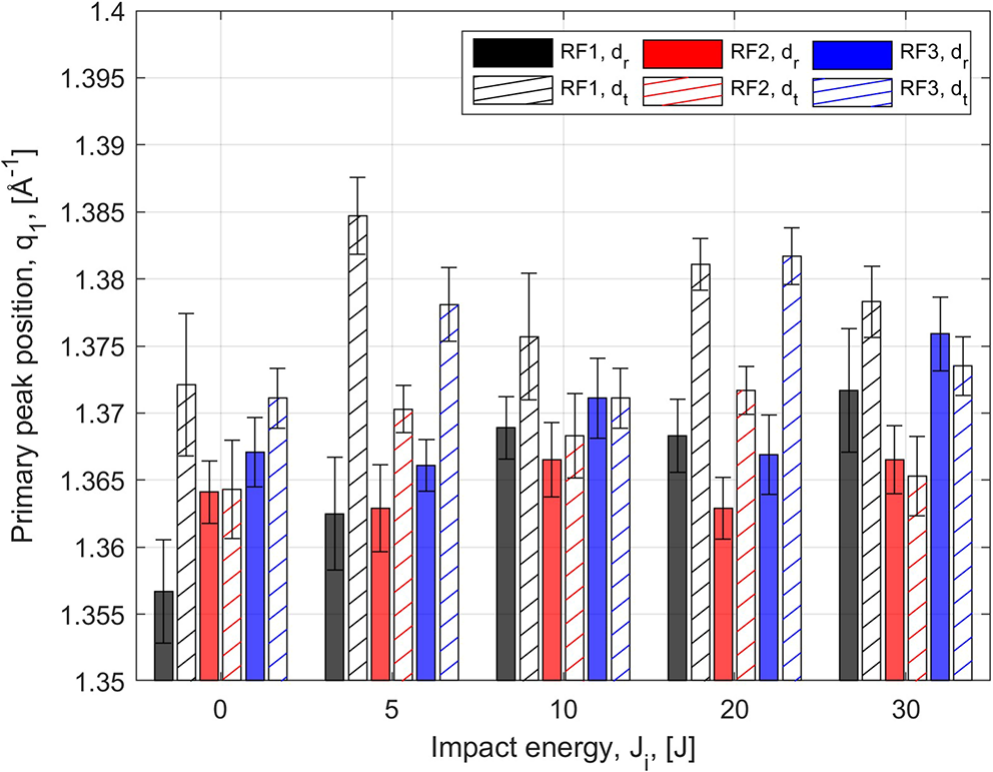
Variation of primary peak location (*q*_1_) with impact energy (*J*_i_). Values obtained via fitting of Gaussian functions to the 1D data of the three foams (RF1, RF2, RF3, see Table 1) in the rise (*d*_r_) and transverse (*d*_t_) direction.

Variations in the peak intensity and position indicate changes in the intermediate-range order of the PU macromolecules. During compression, *q* shifts to higher values, indicating a decrease in the crystalline interplanar distance (*d* = 2π/*q*). In the case of the RPUFs, this phenomenon might be related to the reduction of distance between hard segments in the PU nanomorphology^84,85^ induced by the macroscopic impact deformations. This result further highlights the multiscale and hierarchical nature of RPUFs.

The concept of normalized intensity (*I*) was introduced to compare the experimental and simulation results. From eq 7, the simulated *I* is normalized by the number of atoms in the system (*N* = 15,700 atoms). The samples investigated via WAXS intuitively present a much larger number of atoms. Therefore, the raw intensity (of both experimental and simulated diffractograms) was normalized by its maximum, which corresponds to *I*_1_.

X-ray diffraction (XRD) signals simulated through molecular dynamics and are shown in Figure 8. A clear *q* shift is observed between the initial configuration (*q*_1_ ≈ 1.41 Å^–1^, see Figure 8(a,b)) and the compressed states along *d*_r_ (*q*_1_ ≈ 1.45 Å^–1^, see Figure 8(c,d)) and *d*_*t*_ (*q*_1_ ≈ 1.5 Å^–1^, see Figure 8(e,f)), for both *c*/*a* = 1.5 and *c*/*a* = 2. As already stated, the secondary peak is absent from the simulated XRD diffractograms. Nevertheless, the presence of the primary peak allows for a comparison.

**Figure 8:**
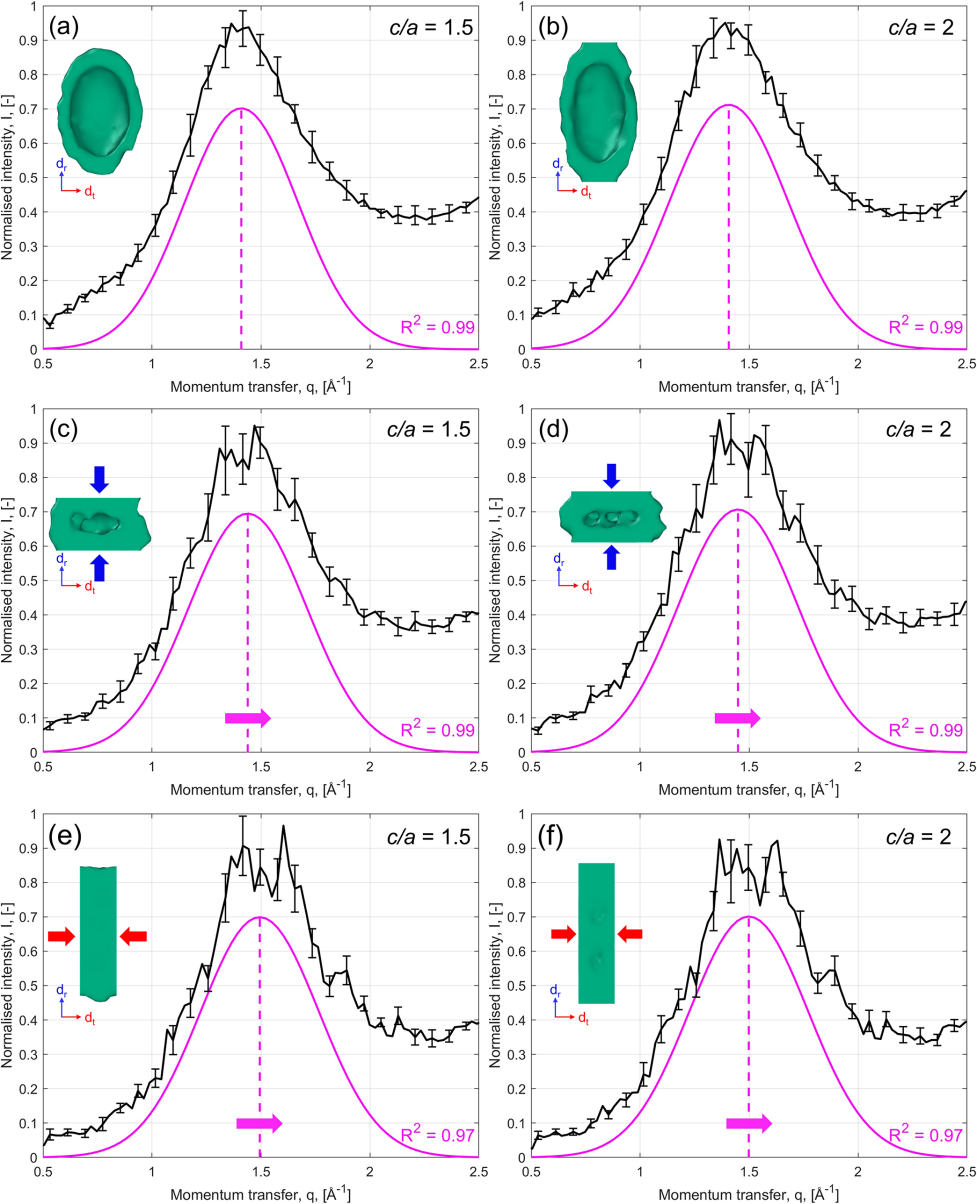
Simulated 1D XRD signals for the pore structures (*c*/*a* = 1.5 left, *c*/*a* = 2 right): (a, b) before compression; (c, d) after compression along the rise (*d*_r_) direction; (e, f) after compression along the transverse (*d*_t_) direction. The primary (magenta) peaks were obtained via fitting of Gaussian functions to the 1D data.

Most of the RPUFs’ mechanical properties (i.e., compressive modulus, yield point and plateau stress) were unaffected by the LVI energy and velocity in the range investigated (see Table 2), as already reported.^42^ On the other hand, the peak stress (σ_max_) and peak strain (ε_max_) depended on the impact conditions. Similarly, the observed *q* shift depends on the value of the applied impact energy. Therefore, nanoscale structural changes reflected in the *q* shift occur primarily at large strains, where cell collapse and densification take place, while small-strain mechanical properties remain largely unaffected.

In addition, it is possible to define a lattice strain (ε_L_) associated with the *q* shift^86^ from eq 10

10 $$\varepsilon_{L}=\frac{d_{f}}{d_{i}}-1$$

In eq 10, *d*_i_ and *d*_f_ are the initial and final crystalline interplanar distance. Although originally defined for crystalline materials, this definition can be extended to amorphous RPUFs to calculate the nanoscopic strain associated with the observed *q* shift resulting from the macroscopic impact process. The relationship between ε_L_, and σ_max_ and ε_max_ is shown in Figure 9, including both experimental and simulation results.

**Figure 9:**
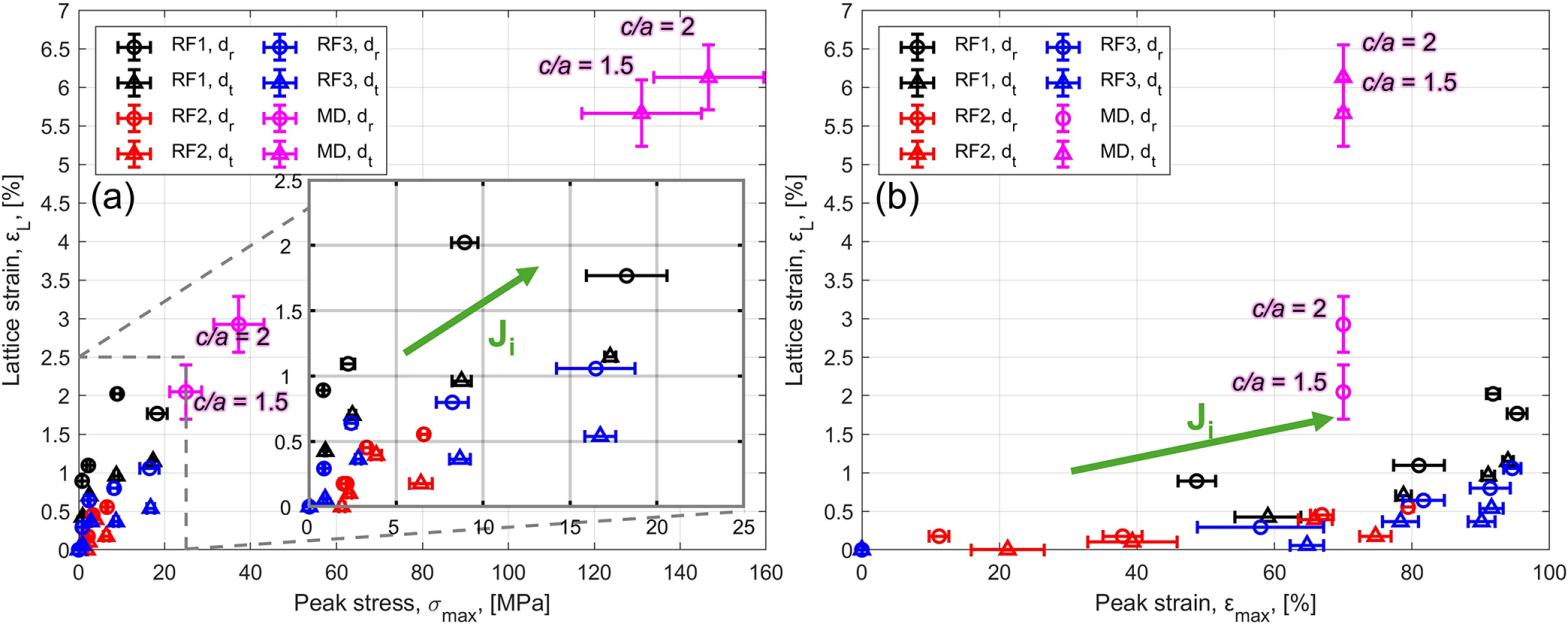
Lattice strain (*ε*_L_) as a function of (a) peak stress (σ_max_) and (b) peak strain (ε_max_). Experimental values for the three foams (RF1, RF2, RF3, see Table 1) and simulated from molecular dynamics (MD, with *c*/*a* = 1.5 and *c*/*a* = 2), in the rise (*d*_r_) and transverse (*d*_t_) direction. Green arrows represent the experimental trends with increasing impact energy (*J*_*i*_).

As expected, based on the mechanical behavior discussed above, the peak stress obtained from MD simulations is much larger than that from experimental measurements, particularly in the transverse direction. Because of the extremely high strain rate employed in the MD simulations (ε̇ = 10^10^ s^–1^) and the isolated nature of the pore, the values of σ_max_ are higher than those measured experimentally in LVI conditions. On the other hand, the peak strain values are comparable between simulations and experiments. Overall, the simulated ε_L_ is always larger than the one provided by experiments, but still with the same order of magnitude. The values for *c*/*a* = 1.5 are lower than those obtained for *c*/*a* = 2 along the rise direction. Conversely, by considering the variability on ε_L_ (from the *q*_1_ 95% fitting confidence intervals) along the transverse direction, the lattice strains are comparable for *c*/*a* = 1.5 and *c*/*a* = 2. Therefore, the transverse response (at the nanoscale) is less sensitive to pore geometry, leading to more similar deformation behavior between the two aspect ratios.

These results further highlight the hierarchical structure of RPUFs, as shown in Figure 10: upon macroscopic compression, the cellular microstructure of the foams is deformed.^42^ In the solid PU polymer, which comprises foam struts and faces of the closed pores, the segmented nanomorphology is altered as hard segments are forced against each other. This reduces their interplanar distance, as reflected in the *q* shift.

**Figure 10:**
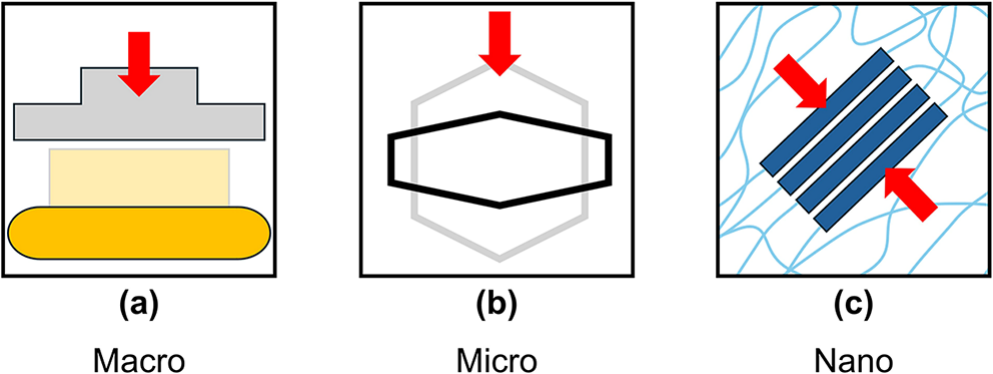
Schematic diagram of the deformation process across length scales in rigid polyurethane foams: (a) at the macro-scale, a rigid impactor compresses a foam specimen (in light yellow) to its deformed configuration (in dark yellow); (b) at the microscale, individual cells (illustrated by a simple hexagon in light gray) are deformed (in black) as a consequence of the macroscopic compression; (c) at the nanoscale, ordered hard segments (in dark blue), immersed in amorphous soft segments (in light blue), are forced against each other because of the deformation of the polyurethane struts, resulting in the *q*_1_ shift discussed above.

The inclusion of multiple closed pores in molecular simulations, alongside a parametric study of the PU chain length and pore dimensions, could improve the agreement with experimental results in the future. The extreme strain rates used in MD simulations are currently not achievable in experimental conditions. Still, further testing at strain rates in the order of 10^4^–10^5^ s^–1^ through Split-Hopkinson pressure bars could further improve the accuracy of the Nagy model and the description of the RPUFs’ mechanical behavior at different strain rates.

## 4 Conclusions

In this work, molecular dynamics simulations and synchrotron wide-angle X-ray scattering were employed to investigate the multiscale structure–property relationships of rigid polyurethane foams. An anisotropic molecular model featuring an ellipsoidal closed pore was implemented, enabling the reproduction of both mechanical response and diffraction behavior. The model successfully captured the presence of an XRD peak at *q* ≈ 1.35 Å^–1^, associated with the hard segment phase at the nanoscale, and its shift upon macroscopic mechanical compression. In addition, the Nagy model was successfully used to bridge the compressive behavior at different strain rates (ε̇) of the foams in experimental (ε̇ = 0.002 s^–1^ and ε̇ = 215 s^–1^) and simulated (ε̇ = 10^10^ s^–1^) conditions.

The observed *q* shift provides insight into the physical mechanisms governing deformation in rigid polyurethane foams. The shift toward higher *q* values indicates a reduction in the interplanar spacing within the hard segment domains, suggesting that macroscopic compression induces nanoscale densification. This finding highlights that the design of more performant polyurethane foams should encompass their segmented nanomorphology, rather than being limited to their cellular architecture at the microscale.

Despite these advances, several limitations remain. The current model should be interpreted as a first-order approximation of the material response at the pore scale, rather than a complete representation of the foam architecture. It considers a single isolated pore, thereby neglecting the interactions within the pore networks of real foams. Additionally, discrepancies between experimental and simulated *q* shifts highlight the role of factors not fully captured in the model, such as viscoelastic relaxation and more complex molecular architectures. In the future, a higher fidelity model would need to be developed to address these limitations. Incorporating multiple interacting pores with varying geometries would enable the study of their effect on stress distribution and structural evolution. More realistic branched polymer architectures representative of biobased polyols, alongside parametric studies of chain length, cross-link density, and pore morphology, would improve fidelity. Future *in situ* SAXS/WAXS experiments will provide further insights at larger length scales,^87,88^ as well as removing potential viscoelastic recovery effects from experimental measurements.

These developments provide a pathway toward resolving current discrepancies and ultimately establishing a predictive, physically grounded model for the design of high-performance polyurethane foams.

## Supplementary Material



## Notes

Detailed information about the Nagy model implementation, SEM images of the foams, and ANOVA tables (PDF)
