# Supplementary material for: X‑rays to Probe Compression across Scales in Rigid Polyurethane Foams: Molecular Simulations and Synchrotron Experiments
Source: ACS Omega. 2026 Apr 27;11(18):27342–54. doi: 10.1021/acsomega.6c01525 (PMC13177015; doi:10.1021/acsomega.6c01525)
Supplement: Supplementary file 1 [file ao6c01525_si_001.pdf]

Supporting Information for:

X-Rays to Probe Compression Across Scales in Rigid Polyurethane Foams: Molecular Simulations and Synchrotron Experiments

Jacopo Lavazza,[*,†] Jula C. Schroeder,[†] Qicheng Zhang,[†,‖] Charles de Kergariou,[†] Oier Bikondoa,[‡,⊥] Jemma L. Rowlandson,[†,#] Tulio Hallak Panzera,[¶] Wuge H. Briscoe,[§] and Fabrizio Scarpa[†]

†*Bristol Composites Institute, University of Bristol, Bristol BS8 1TR, UK*

‡*XMaS, The UK CRG Beamline, European Synchrotron Radiation Facility (ESRF), Grenoble 38043, France*

¶*Centre for Innovation and Technology in Composite Materials, Department of Mechanical and Production Engineering, Federal University of São João del Rei, São João del Rei 36301-158, Minas Gerais, Brazil*

§*School of Chemistry, University of Bristol, Bristol BS8 1TS, UK*

‖*School of Energy and Power Engineering, Beihang University, Beijing 100083, China*

⊥*Department of Physics, University of Warwick, Coventry CV4 7AL, UK*

#*School of Electrical, Electronic and Mechanical Engineering, University of Bristol, Bristol BS8 1TR, UK*

E-mail: jacopo.lavazza@bristol.ac.uk

S1. The Nagy model

The Nagy model was used to predict the material response at the extremely high strain rates ($\dot{\varepsilon}$) used during molecular dynamics (MD) simulations ($\dot{\varepsilon} = 10^{10}$ s^{-1}). The mean experimental curves for quasi-static (QS, $\dot{\varepsilon} = 0.002$ s^{-1}) and low-velocity impact (LVI, $\dot{\varepsilon} = 215$ s^{-1}) compression were employed to calibrate the model parameters (a and b), reported in Table S1. Relevant experimental mechanical properties are reported in Table S2 and Table S3 for QS and LVI conditions, respectively. Small differences between Table S2 and Table S3 (e.g., ε_d) are within experimental variability ($\leq 5\%$) and are not considered significant.

Table S1: Adimensional fitting parameters (a and b) of the Nagy model for the three foams (RF1, RF2, RF3) in the rise (d_r) and transverse (d_t) direction.

Foam	Direction	a, [-]	b, [-]
RF1	d_r	-0.062	0.032
	d_t	-0.034	0.034
RF2	d_r	-0.037	0.065
	d_t	-0.01	0.07
RF3	d_r	-0.051	0.038
	d_t	-0.01	0.029

Table S2: Mean mechanical properties obtained from the quasi-static curves at strain rate $\dot{\varepsilon} = 0.002$ s^{-1} for the three foams (RF1, RF2, RF3) in the rise (d_r) and transverse (d_t) direction. Compressive modulus (E), yield stress (σ_y), plateau stress (σ_p), yield strain (ε_y) and densification strain (ε_d).

Foam	Direction	E, [MPa]	σ_y, [MPa]	σ_p, [MPa]	ε_y, [%]	ε_d, [%]
RF1	d_r	9.4	0.39	0.56	4.3	50.8
	d_t	6.4	0.26	0.45	4.2	51.5
RF2	d_r	12.5	0.58	0.95	5.3	52.1
	d_t	8	0.34	0.65	4.7	47.3
RF3	d_r	6.6	0.33	0.44	5.3	50.6
	d_t	4	0.19	0.36	5.2	51.1

The model's predictive ability was verified for LVI conditions, and relevant mechanical properties are reported in Table S4. As shown in Figure S1 and Table S4, the correlation coefficients (R^2) between the experimental and predicted LVI curves are all above 0.95.

Table S3: Mean mechanical properties obtained from the low-velocity impact curves at strain rate $\dot{\varepsilon} = 215$ s^{-1} for the three foams (RF1, RF2, RF3) in the rise (d_r) and transverse (d_t) direction. Compressive modulus (E), yield stress (σ_y), plateau stress (σ_p), yield strain (ε_y) and densification strain (ε_d).

Foam	Direction	E, [MPa]	σ_y, [MPa]	σ_p, [MPa]	ε_y, [%]	ε_d, [%]
RF1	d_r	12.5	0.51	0.66	4.4	50.1
	d_t	8	0.39	0.6	5.6	50.7
RF2	d_r	20.7	1.4	1.9	8.1	55.7
	d_t	21.9	1.5	2.2	8.2	54.8
RF3	d_r	12,2	0.45	0.52	3.6	46.3
	d_t	5.4	0.27	0.46	5.5	48.3

Table S4: Mean mechanical properties, correlation coefficients (R^2) and root mean square errors ($RMSE$) obtained from the Nagy model at strain rate $\dot{\varepsilon} = 215$ s^{-1} for the three foams (RF1, RF2, RF3) in the rise (d_r) and transverse (d_t) direction. Compressive modulus (E), yield stress (σ_y), plateau stress (σ_p), yield strain (ε_y) and densification strain (ε_d).

Foam	Direction	R^2, [-]	$RMSE$, [-]	E, [MPa]	σ_y, [MPa]	σ_p, [MPa]	ε_y, [%]	ε_d, [%]
RF1	d_r	0.99	0.05	13.1	0.55	0.67	4.4	52.6
	d_t	0.99	0.04	9.4	0.38	0.59	4.3	52.5
RF2	d_r	0.97	0.25	25.8	1.2	1.8	5.3	53.2
	d_t	0.95	0.63	17.9	0.76	1.4	4.7	47.3
RF3	d_r	0.97	0.05	9.9	0.48	0.57	5.3	52.2
	d_t	0.99	0.05	5.5	0.26	0.48	5.3	51.3

In addition, the standardised residuals (s) are reported in Figure S2 and only show poor predictions during the densification stage ($\varepsilon > 60\%$), which is a known limitation of the model. The root mean square errors ($RMSE$, see Table S4) are ≤ 0.05 for RF1 and RF3, while higher for RF2, particularly along d_t, as the predicted curves show more pronounced deviations from experimental data.

The standardised residuals are defined in Equation (S1):

$$s(\varepsilon) = \frac{\sigma_{Exp}(\varepsilon) - \sigma_{Pre}(\varepsilon)}{std(\sigma_{Exp} - \sigma_{Pre})}, \tag{S1}$$

where $\sigma_{Exp}(\varepsilon)$ and $\sigma_{Pre}(\varepsilon)$ are the experimental and predicted LVI stress, respectively.

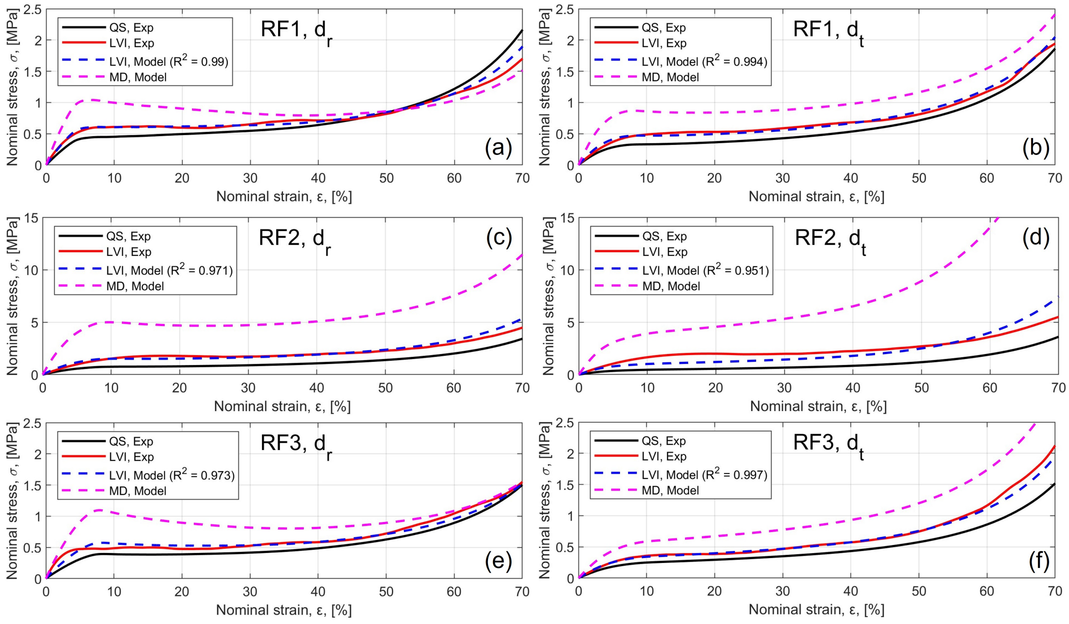

Figure S1: Nominal stress-strain ($\sigma - \varepsilon$) curves at different strain rates ($\dot{\varepsilon}$) in the rise (d_r) and transverse (d_t) direction for (a-b) RF1, (c-d) RF2, (e-f) RF3. Experimental (Exp) curves for quasi-static (QS, $\dot{\varepsilon} = 0.002$ s^{-1}) and low-velocity impact (LVI, $\dot{\varepsilon} = 215$ s^{-1}) compression. Predictions (Model) of the Nagy model in LVI ($\dot{\varepsilon} = 215$ s^{-1}) and molecular dynamics (MD, $\dot{\varepsilon} = 10^{10}$ s^{-1}) conditions.

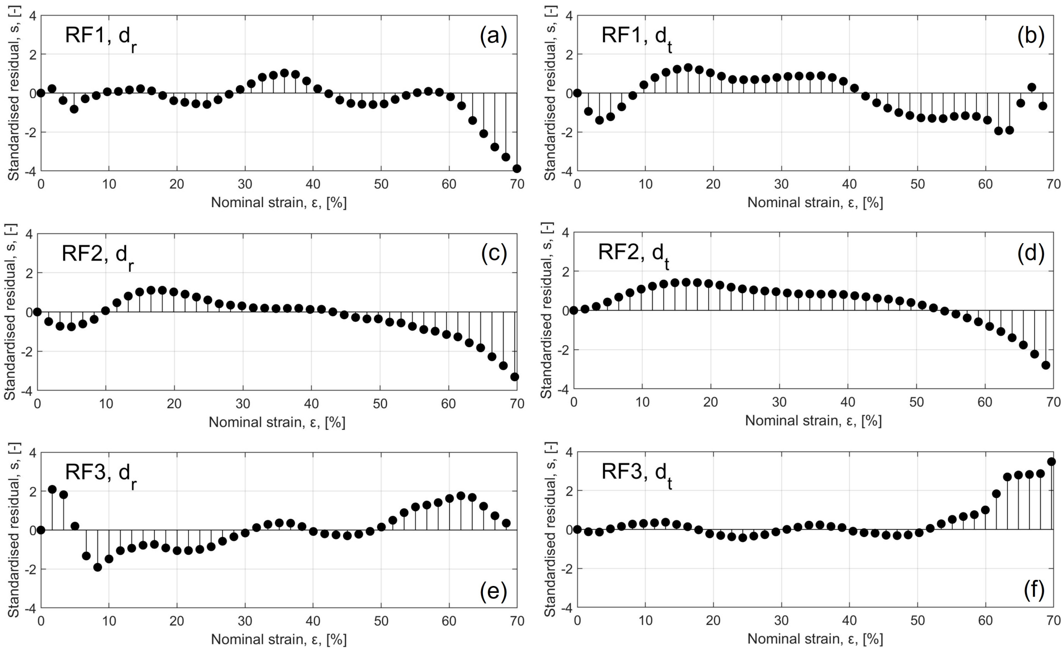

Figure S2: Standardised residual-strain ($s - \varepsilon$, see Equation (S1)) plots obtained from the low-velocity impact (LVI, $\dot{\varepsilon} = 215$ s^{-1}) experimental and predicted curves in Figure S1 in the rise (d_r) and transverse (d_t) direction for (a-b) RF1, (c-d) RF2, (e-f) RF3.

S2. Determination of the c/a ratio

The pore anisotropy degree was identified by the ellipsoidal axes ratio (c/a). Experimentally, the ratio was determined from scanning electron microscopy (SEM) images of untested foam samples, reported in Figure S3. Imaging was performed on a Hitachi TM3030Plus tabletop microscope under a high vacuum, operated at an acceleration voltage of 15 kV and at an operating distance of approximately 5.8 mm. Microscopy was performed on cubic specimens of about $5 \times 5 \times 5$ mm^3 in size. Quantitative analysis was performed in the Fiji/ImageJ software by fitting ellipses to the pores in Figure S3 and determining their c/a ratio. The pores are elongated along the foam rise direction (d_r) for all three foams. The obtained values are 1.9 ± 0.4 for RF1, 1.6 ± 0.2 for RF2 and 1.5 ± 0.3 for RF3.

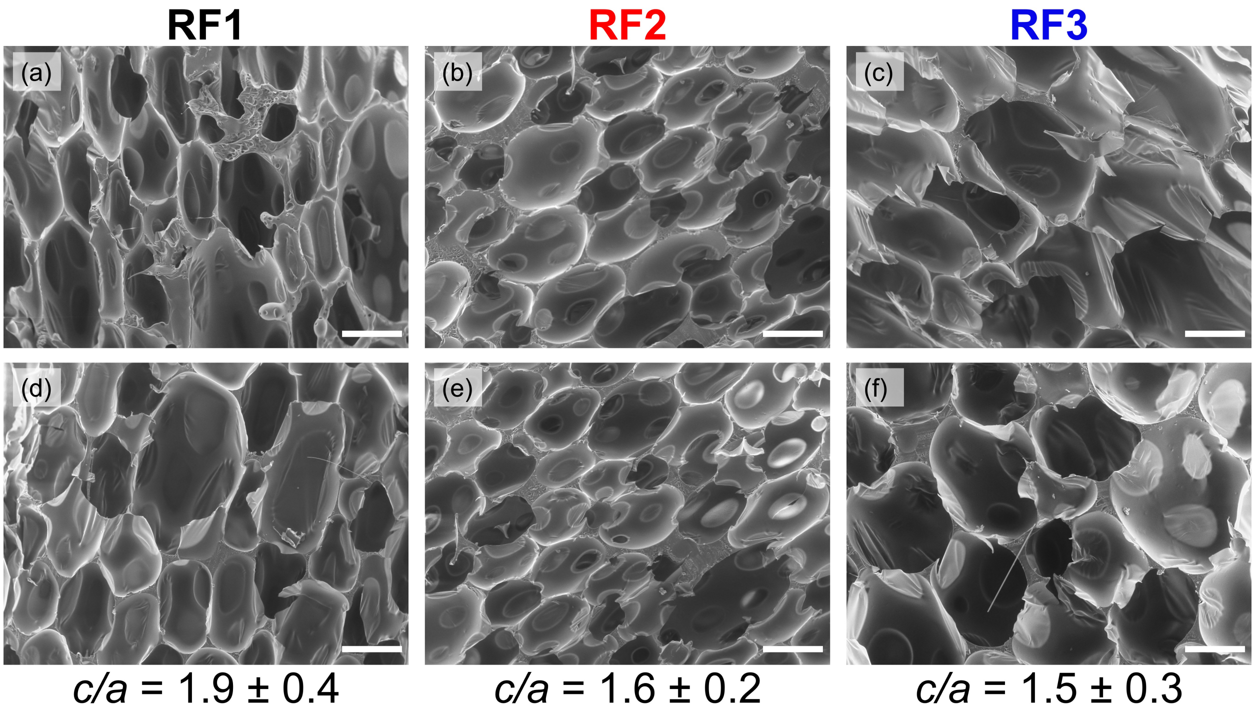

Figure S3: Scanning electron microscopy (SEM) images of untested foam samples and measured ellipsoidal axes ratio (c/a): (a-c) RF1, (b-e) RF2, (c-f) RF3. The scale bars refer to 500 μm.

S3. ANOVA tables for I_1 comparison

Two-way analysis of variance (ANOVA) was performed to evaluate the effects of impact energy (J_i) and loading direction (d_r and d_t), as well as their interactions, on the primary peak intensity (I_1) of the three foams. The ANOVA tables are reported in Tables S5 to S7 for RF1, RF2 and RF3, respectively.

Table S5: ANOVA table for the primary peak intensity (I_1) of RF1.

Source	SS	df	MS	F	p
Columns (d)	1.8587×10^7	1	1.8587×10^7	9.40	0.0026
Rows (J_i)	7.6884×10^7	4	1.9221×10^7	9.72	< 0.001
Interaction	1.3647×10^8	4	3.4118×10^7	17.26	< 0.001
Error	2.7680×10^8	140	1.9771×10^6		
Total	5.0874×10^8	149			

Table S6: ANOVA table for the primary peak intensity (I_1) of RF2.

Source	SS	df	MS	F	p
Columns (d)	4.8359×10^6	1	4.8359×10^6	1.41	0.2363
Rows (J_i)	2.1349×10^8	4	5.3373×10^7	15.61	< 0.001
Interaction	1.2105×10^7	4	3.0263×10^6	0.89	0.4747
Error	4.7867×10^8	140	3.4191×10^6		
Total	7.0911×10^8	149			

Table S7: ANOVA table for the primary peak intensity (I_1) of RF3.

Source	SS	df	MS	F	p
Columns (d)	7.8751×10^4	1	7.8751×10^4	0.03	0.8556
Rows (J_i)	2.2397×10^8	4	5.5994×10^7	23.63	< 0.001
Interaction	2.7510×10^7	4	6.8776×10^6	2.9	0.0241
Error	3.3174×10^8	140	2.3695×10^6		
Total	5.8330×10^8	149			

S4. ANOVA tables for q_1 comparison

Two-way analysis of variance (ANOVA) was performed to evaluate the effects of impact energy (J_i) and loading direction (d_r and d_t), as well as their interactions, on the primary peak position (q_1) of the three foams. The ANOVA tables are reported in Tables S8 to S10 for RF1, RF2 and RF3, respectively.

Table S8: ANOVA table for the primary peak position (q_1) of RF1.

Source	SS	df	MS	F	p
Columns (d)	0.00577	1	0.00577	4.24	0.0413
Rows (J_i)	0.87558	4	0.2189	161.02	< 0.001
Interaction	0.08648	4	0.02162	15.9	< 0.001
Error	0.19032	140	0.00136		
Total	1.15815	149			

Table S9: ANOVA table for the primary peak position (q_1) of RF2.

Source	SS	df	MS	F	p
Columns (d)	0.03557	1	0.03557	48.04	< 0.001
Rows (J_i)	0.07514	4	0.01879	25.37	< 0.001
Interaction	0.01258	4	0.00314	4.25	0.0028
Error	0.10368	140	0.00074		
Total	0.22697	149			

Table S10: ANOVA table for the primary peak position (q_1) of RF3.

Source	SS	df	MS	F	p
Columns (d)	0.09526	1	0.09526	153.07	< 0.001
Rows (J_i)	0.23458	4	0.05864	94.24	< 0.001
Interaction	0.02318	4	0.00580	9.31	< 0.001
Error	0.08712	140	0.00062		
Total	0.44014	149			
